# Evaluation of Anti-Glycopeptidolipid-Core Immunoglobulin A Antibody Detection for the Diagnosis of Nontuberculous Mycobacterial Cervicofacial Lymphadenitis

**DOI:** 10.1097/INF.0000000000004462

**Published:** 2024-06-26

**Authors:** Samuel H. Willemse, Marjolijn A.E.M. Oomens, Luc H.E. Karssemakers, Jerome A. Lindeboom, Willem H. Schreuder, Jean-Pierre T.F. Ho, Martijn van der Kuip, Killian E. Vlaming, Tanja M. Kaptein, Jan de Lange

**Affiliations:** * From the Department of Oral and Maxillofacial Surgery, Amsterdam University Medical Center (UMC) and Academic Centre for Dentistry Amsterdam, University of Amsterdam, Amsterdam, the Netherlands; † Department of Oral and Maxillofacial Surgery, Rijnstate Hospital, Arnhem, the Netherlands; ‡ Department of Head and Neck Oncology and Surgery, Netherlands Cancer Institute/Antoni Van Leeuwenhoek Hospital, Amsterdam, the Netherlands; § Department of Oral and Maxillofacial Surgery, Amstelland Hospital, Amstelveen, the Netherlands; ¶ Department of Oral and Maxillofacial Surgery, Northwest Clinics, Alkmaar, the Netherlands; ∥ Department of Pediatric Infectious Diseases and Immunology, Amsterdam UMC, Emma Children’s Hospital, University of Amsterdam, Amsterdam, the Netherlands; ** Department of Experimental Immunology, Amsterdam UMC, University of Amsterdam, Amsterdam, the Netherlands; †† Amsterdam Institute for Infection and Immunity, Amsterdam, the Netherlands.

**Keywords:** nontuberculous, lymphadenitis, serodiagnosis

## Abstract

A multicenter cross-sectional diagnostic study was carried out including 45 children with nontuberculous mycobacterial cervicofacial lymphadenitis and controls. The tested immunoassay, detecting *M. avium–*specific anti-glycopeptidolipid-core immunoglobulin A antibodies, had inadequate diagnostic performance in the studied population and seems to be of no additional value in detecting cases of nontuberculous mycobacterial cervicofacial lymphadenitis.

## INTRODUCTION

A timely diagnosis of nontuberculous mycobacterial (NTM) cervicofacial lymphadenitis in children is important to optimize treatment outcomes.^1^ An enzyme immunoassay measuring serum IgA (immunoglobulin A) antibodies that bind *Mycobacterium avium* complex (MAC) specific anti-glycopeptidolipid (GPL)-core antigens is currently commercially available. MAC is a serological complex of serovars of *M. avium* and *M. intracellulare*.^2^ These account for approximately 75% of NTM cervicofacial lymphadenitis cases in children in the Netherlands and are predominantly identified as causative agents globally.^3,4^ The present study aimed to investigate the diagnostic performance of the detection of MAC-specific anti-GPL-core IgA antibodies in serodiagnosis of NTM cervicofacial lymphadenitis.

## MATERIALS AND METHODS

### Subjects

A multicenter cross-sectional study was carried out in the Amsterdam University Medical Center (UMC) and Radboud UMC. Consecutively presenting children (0–15 years of age) who underwent surgery under general anesthesia because of a strong clinical suspicion of NTM cervicofacial lymphadenitis were eligible for enrollment. Clinical suspicion was based on cervicofacial lymphadenopathy persisting for a period of at least 3 weeks, negative serologic tests for other causes of (sub)chronical lymphadenopathy (Epstein-Barr virus, cytomegalovirus, Bartonella species and *Toxoplasma gondii*) and suspicious sonographic features (hypoechoic lymph nodes, often with central necrosis, nodal matting and adjacent soft tissue edema).^5^

A control group comprised children (0–15 years of age) undergoing surgery under general anesthesia for reasons other than lymphadenopathy.

### Microbiologic Laboratory Procedures

Microbiologic analysis of excised lymph node and pus samples of patients suspected of NTM cervicofacial lymphadenitis included an *M. tuberculosis* polymerase chain reaction (PCR), mycobacterial genus PCR, *M. avium* complex PCR, mycobacterial culture, auramine/Ziehl Neelsen staining, *B. henselae* PCR and a conventional microbial culture. Histopathological examination was also performed.

### Serum Analysis

Blood samples were taken during general anesthesia and prior to the start of the surgical procedure. The serum was isolated subsequently and stored at −80 °C and thawed upon testing. Serum IgA antibodies against the GPL-core antigen were measured using an enzyme immunoassay kit (Tauns Laboratory, Inc, Shizuoka, Japan) according to the manufacturer’s instructions. Results were given as U/mL, and a cutoff value of 0.7 U/mL was used as a threshold to differentiate between positive and negative as advised by the manufacturer.

### Study Groups

Subjects were divided into 3 groups.

1. Proven NTM lymphadenitis based on microbiological confirmation by PCR, culture or acid-fast staining techniques.
2. Probable NTM lymphadenitis lacking microbiological confirmation. The probable diagnosis was based on cervicofacial lymphadenopathy for a period of at least 3 weeks, suspicious sonographic features (hypoechoic lymph nodes, often with central necrosis, nodal matting and adjacent soft tissue edema) and serologic exclusion of other infectious causes of (sub)chronic lymphadenopathy and corresponding histopathologic results (microabscesses, necrotizing granulomas and giant cells).^5,6^
3. Controls without any history of suspicion or clinical signs of a past or current NTM cervicofacial lymphadenitis.

If postoperative microbiological and histopathologic examination revealed a confirmed cause of lymphadenopathy other than NTM cervicofacial lymphadenitis, patients were classified as controls.

### Ethical Approval

The study was formally approved by the Medical Ethics Review Committee of the Amsterdam UMC, location AMC (reference number 2018_315), and the boards of directors of the Amsterdam UMC and Radboud UMC.

## RESULTS

In total, 45 subjects were enrolled in this study between October 2019 and November 2023, 32 of whom underwent surgery because of a strong clinical suspicion of NTM cervicofacial lymphadenitis. Of these 32 patients, 22 NTM cases were microbiologically confirmed postoperatively with either culture, PCR or acid-fast staining techniques. The identifying species are demonstrated in the Table. In 5 cases, histopathologic examination revealed (necrotizing) granulomatous infection suggestive of mycobacterial disease, but microbiologic confirmation with PCR, culture or staining techniques was not achieved.

**TABLE.** Subject Characteristics

	Microbiologically confirmed NTM lymphadenitis (N = 22)	Probable NTM lymphadenitis (N = 5)	Controls (N = 18)
Sex, n (%)			
Male	10 (45)	3 (40)	9 (50)
Female	12 (55)	2 (60)	9 (50)
Mean age at time of sampling in years ± SD (range)	2.9 ± 1.5 (1.5–6.2)	3.7 ± 3.4 (1.1–9.7)	4.3 ± 4.0 (1.0–16.0)
Mean time from first symptoms to date of sampling in weeks ± SD (range)	15 ± 23 (3–114)	35 ± 31 (4–73)	
Causative species, n (%)			
Mycobacterium avium complex	17 (77)		
*M. avium*	16 (73)		
*M. florentinum*	1 (5)		
*M. haemophilum*	1 (5)		
*M. chimaera*	1 (5)		
*M. kansassi*	1 (5)		
*M. malmoense*	1 (5)		
Unspecified	1 (5)		

M indicates mycobacterium; N, number of included subjects; and NTM, nontuberculous mycobacterial.

The other 5 patients turned out to have different diagnoses (*B. henselae* lymphadenitis, group A *Streptococcus* lymphadenitis, methicillin-resistant *S. aureus* lymphadenitis, ruptured epidermoid cyst and suppurative lymphadenitis without identified species) than previously suspected and were classified as controls. An additional 13 patients without any history of clinical suspicion or signs of a past or current NTM cervicofacial lymphadenitis were included in the control group. The Table summarizes the characteristics of the included subjects.

The sensitivity was 36%, the specificity was 83% (N = 40 [95% confidence interval (CI), 17%–35% and 59%–96%]), and the area under the curve was 0.55 [N = 40 (95% CI, 0.37–0.73)]. An area under the curve of 0.53 [N = 39 (95% CI, 0.33–073)] was found in a subgroup analysis evaluating MAC lymphadenitis and controls.

## DISCUSSION

The current study evaluated the diagnostic accuracy of an enzyme immunoassay kit measuring MAC-specific anti-GPL-core IgA antibodies in children with and without NTM cervicofacial lymphadenitis. Although some microbiologically proven patients had a positive titer, the test lacked adequate sensitivity and specificity. Subgroup analyses revealed no additional diagnostic value of the MAC-specific anti-GPL-core IgA antibodies assay for subjects with NTM lymphadenitis caused by MAC.

As far as the authors are aware, this is the first study to systematically investigate this immunoassay in subjects with NTM cervicofacial lymphadenitis. The value of the area under the curve found in this study (0.55) is lower than the area under the curve reported in a systematic review from 2016 by Shibata et al^7^ (0.87), which focused on the diagnostic test accuracy for MAC pulmonary disease. This discrepancy indicates a comparatively poorer diagnostic performance of the tested assay in patients with MAC cervicofacial lymphadenitis.^7^ Patients with NTM cervicofacial lymphadenitis are in contrast to MAC pulmonary disease not immunocompromised and are also generally younger. Pulmonary infections include larger infected tissue surfaces and the immunogenic pathway of MAC cervicofacial lymphadenitis might be different from MAC pulmonary disease. Children are known to have a more immature cellular immunity compared to adults, and IFN-gamma release of NTM cervicofacial lymphadenitis patients has been demonstrated to be impaired after mitogenic or antigenic stimulation during their infection.^8^ It could be hypothesized that patients with NTM cervicofacial lymphadenitis exhibit increased salivary IgA levels instead of increased serum IgA levels due to the location of the infection in the head and neck area and the absence of general disease symptoms.^9^ Moreover, none of the included studies in the systematic review of Shibata et al was carried out in a European center, reflecting heterogeneity in the included study subjects compared with those in the current study.

Immunodiagnostic assays with PPD stimulation are a promising noninvasive diagnostic alternative. Recently, an NTM-specific interferon-γ release assay, stimulated by GPLs, was investigated. The sensitivity and specificity estimates of this immunodiagnostic assay were 92% and 100%, respectively.^10^

## CONCLUSIONS

The tested enzyme immunoassay measuring MAC-specific anti-GPL-core IgA antibodies has inadequate diagnostic performance in the studied population of patients with NTM cervicofacial lymphadenitis in contrast to those with MAC pulmonary disease and seems to be of no additional value in detecting cases of NTM cervicofacial lymphadenitis. To optimize outcomes in these patients with NTM cervicofacial lymphadenitis, further research on (relatively) noninvasive methods for early detection is needed.
